# Upright and inverted unfamiliar face-matching tasks – everything correlates everywhere all at once

**DOI:** 10.3758/s13421-025-01725-w

**Published:** 2025-05-07

**Authors:** Jeremy J. Tree, Alex L. Jones, Robin S. S. Kramer

**Affiliations:** 1 School of Psychology, Faculty of Medicine, Health and Life Sciences, University of Swansea, Swansea, SA2 8PP UK; 2 School of Psychology, Sport Science and Wellbeing, University of Lincoln, Lincoln, UK

**Keywords:** Face processing, Face matching, Face inversion effects, Psychometrics

## Abstract

In a key study, Megreya and Burton (*Memory & Cognition*, *34*, 865–876, 2006) argued that identity-matching tasks using unfamiliar faces may not effectively measure general ‘real-world’ face-processing ability – that is they are “not faces”. They observed a high correlation in performance between upright and inverted unfamiliar face matching, a pattern not seen with familiar faces, which they interpreted as indicating unfamiliar face matching is *qualitatively* different and largely driven by image-specific factors. However, the authors cautioned that this limitation likely applies only to unfamiliar face-matching tasks for *identity* rather than other types of face judgements (e.g., emotion). The present study replicates and extends these findings by considering within-subject performance for upright/inverted unfamiliar face matching across various paradigms (sequential/simultaneous presentation or sorting) and face-judgement types (identity or emotion), whilst considering different types of measures (accuracy and reaction time). Our results illustrated high correlations for upright/inverted conditions were universally observed *within* tasks for both accuracy and reaction times. Subsequent factor analyses indicated that upright and inverted conditions loaded together into *task-specific* latent variables. These results concur with the conclusions of Megreya and Burton (2006) and extend to both identity and emotion matching tasks – that is such tasks exhibit low *construct validity* for testing hypotheses about much general ‘everyday’ face processing. We propose that researchers should carefully consider alignment between their test materials and the theoretical ‘constructs’ they aim to measure, ensuring more accurate and meaningful interpretations of their results.

## Introduction

Researchers have become more and more interested in exploring the degree of individual differences observed in face-processing ability, since the initial assumptions that human beings are likely universally ‘expert’ face recognisers (e.g., Diamond & Carey, 1986) have proven quite wrong. The fact that this type of assumption was so pervasive in the field for so long is perhaps unsurprising – if one assumes that face-recognition performance draws on a long history of natural selection, it intuitively follows that individual variability would probably be quite small. However, it has been repeatedly observed that scores for individuals on standardised measures of face recognition (such as the Cambridge Face Memory Test (CFMT); Duchaine & Nakayama, 2006), yield distributions of performance that range from what would be considered quite poor to outstandingly good. Individuals at the ‘extremes’ of this distribution of performance have since been given their own names; developmental prosopagnosia (DP*)* for those doing very poorly (e.g., Bate et al., 2019a, b; Behrmann & Avidan, 2005; Bennetts et al., 2022) and super-recognisers (SR) for those doing very well (e.g., Bobak et al., 2016; Davis et al., 2016; Ramon, 202).

As a consequence, alongside the growing interest in identifying, and thus subsequently studying, individuals at the population ‘extremes’, there has been an emerging industry around developing face-based tests that can perhaps ‘diagnose’ individuals as being DP or SR. In particular, with an apparent focus on face-perception testing, there has been a growth of new unfamiliar face-*matching* tasks, such as The Kent Face Matching Test (KFMT; Fysh & Bindemann, 2018), the Expertise in Facial Comparison Test (EFCT; White et al., 2015), the 1 in 10 test (Bruce et al., 1999), the Person Identification Challenge Test (PICT; White et al., 2015), the Face Identity Card Sorting Test (FICST; Stacchi et al., 2020), the Glasgow Face Matching Test (GFMT; Burton et al., 2010), the Benton facial recognition test (Murray et al., 2022) and the Glasgow Face Matching Test 2 (GFMT2; White et al., 2022). Although at their heart all these tests are asking participants the same basic question, namely *are these two faces the same person*?, specific paradigm elements can vary considerably. Including the basic procedure for stimulus presentation (e.g., simultaneous presentation, sequential presentation or sorting), the stimulus format (e.g., images varying in lighting, pose, etc.) and even the response format (Yes/No or Likert scale). A potential key implicit assumption is that participant performance on such tests is a ‘window’ into how individuals do with everyday (familiar) face recognition – that is, the tests are a valid proxy of this *latent ability*. The current study investigates whether these tasks truly capture a singular ‘construct’ of ‘real-world’ face-processing ability – some *general* face-perception factor (akin to “f”; Verhallen et al., 2017) – by building on earlier work by Megreya and Burton (2006) that questions this assumption. But before moving onto the specifics of the current work, it is important to set the scene by discussing two key issues: (a) how test *construct validity* is typically interpreted, drawing on the venerable work of psychometrics, and (b) why the face-inversion effect was used to challenge this validity assumption.

## Unfamiliar face-matching tasks – are they a valid measure of latent face ability?

In the field of psychometrics, it has long been the goal to create psychological tests that can measure aspects of human performance of a variety of types – and a key concept that is relevant here is construct validity which refers to a simple question: does this instrument really probe the underlying *functional process* (theoretical concept) of interest? Namely, is it really tapping my day-to-day latent ability at recognising faces in the ‘real world’? Clearly the implications of tests that are poor in construct validity are simple: they are not appropriate for making any inferences about how the human face-processing system works. In the case of unfamiliar face-matching tasks, two key pieces of evidence give us reason for concern. First, since these matching tasks essentially ask participants to decide if two images of an unfamiliar person constitute the same person*,* we might ask how much performance varies if one systematically manipulates the properties of such images. Unfortunately, there is worrying evidence that performance is image bound*,* in that ability has been observed to drop dramatically if there are even small changes in the angle of presentation (e.g., Longmore et al., 2008; Megreya et al., 2013), whilst conversely ability has been observed to *improve* when participants are instructed to focus on specific features of images presented (Megreya, 2018; Towler et al., 2021; though also see Kramer, 2023). That is, good performance seems very much dependent on the *specific image* presented and even boosted by guidance to look for *key features* in such images. Moreover, test–retest reliability of face-matching tasks (i.e., individual scores across two testing time periods), a key measure of test *reliability*, is also observed to be poor, “*despite apparent item homogeneity*” (Petersen & Leue, 2022). This image-specific performance, which is not even stable across time, has been interpreted as suggesting individual participants may be undertaking unfamiliar face-matching tasks by adopting idiosyncratic paradigm-specific heuristics (Dunn et al., 2024). Bruce et al. (1999) and Hancock et al. (2000) have suggested unfamiliar face matching may be better thought of as simple image matching, rather than involving more general face-processing systems. Consequently, observed performance by participants will have little to do with face processing in the ‘real world’ (see Goodhew & Edwards, 2019, for a related more general discussion)*.*

**Fig. 1:**
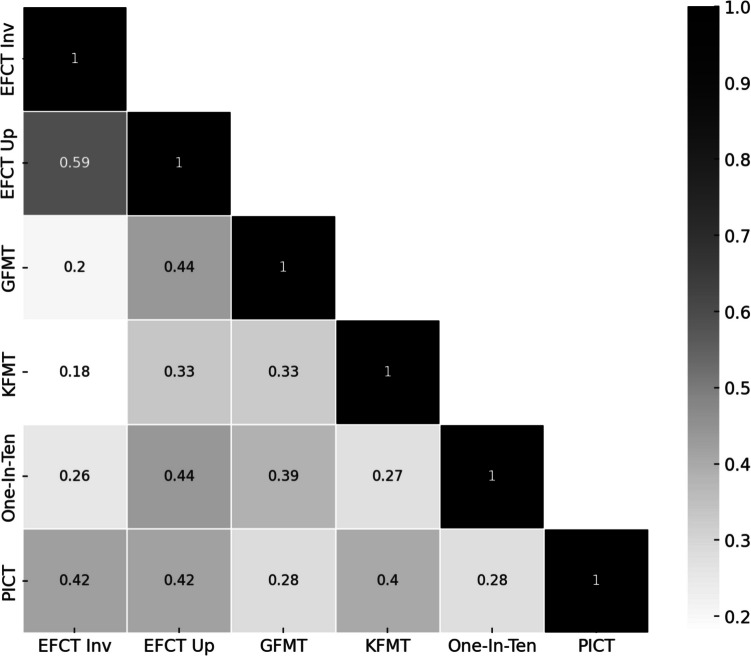
Correlations between five key unfamiliar face-matching tasks (Bobak et al., 2023)

Second, an additional approach adopted by psychometrics researchers is to explore construct validity by correlating performance *across* similar related tasks (see Kramer & Tree, 2024, and Wilmer, 2008), where it is expected tasks that tap the same process or ‘construct’ (i.e., one’s latent ability at ‘everyday’ face processing) will have high correlations (*convergent validity*), and other tasks that do not tap the same process will have low correlations (*discriminant validity*). Unfortunately, with respect to convergent validity, an emerging body of work has observed poor correlations between unfamiliar face-matching tasks. Figure 1 shows the correlation matrix reported by Bobak et al. (2023), where the researchers investigated cross-task performance across five lab-based unfamiliar face-matching tasks. The range of these correlations for psychometric purposes is often low (0.44 to 0.18) – the highest being a *within-task* correlation of upright/inverted trials on the EFCT (0.59), which is relevant for a later point. Other work has similarly observed low cross-task correlations (e.g., Fysh & Bindeman, 2018 (GFMT/KFMT *r* = 0.45); Fysh et al., 2020 (One-in-Ten/KFMT; *r* = −0.31); see also Burton & Jenkins, 2011, for discussion). Moreover, although the focus of this study is on face matching, similarly low correlations have been observed when measuring performance on unfamiliar face *recognition* memory paradigms to matching tasks (e.g., Verhallen et al., 2017).

Reflecting on their findings, Bobak and colleagues write *“Extending previous work (*Bate et al., 2018; Fysh et al., 2020; Stacchi et al., 2020*), our results revealed small to medium correlations between tests and thus large individual differences between them, even when operating within the same paradigm (simultaneous matching tasks). The intraclass correlation coefficient (ICC) (below 0.40), further confirmed low consistency in performance (i.e. large variability in ranking on different tests between participants) in our sample”.* This is despite the fact that these same authors point out the five key tests under consideration are “*nearly procedurally identical”* – with a key difference often being about the *particular stimuli* employed – the GFMT images were taken on the same day, with two different cameras; the KFMT images were taken months apart, with different devices and at varying distances; and the EFCT/PICT images were taken at varying distances, in different lighting and over a period of 2 years (Bobak et al., 2023; Phillips et al., 2011). These findings suggest that low convergent validity may be driven by participants’ reliance on idiosyncratic, image-specific heuristics – people appear to use varied, task- and trial-specific strategies in each face-matching test (Dunn et al., 2024), leading to lower cross-task correlations (Fysh et al., 2020). This issue raises concerns about construct validity: if unfamiliar face-matching performance is shaped primarily by *test-specific* procedures and stimuli, it may not accurately reflect the broader ‘construct’ of more general face processing. As we will discuss, the principle of convergent/discriminant validity hinges on how we interpret the magnitude of correlations. But it appears at this point that we may have reason to question whether key unfamiliar face-matching tasks genuinely tap some general ‘construct’ of face-recognition skill. That is, if results across similar face-matching tests vary widely, perhaps performance is driven more by stimulus-specific, idiosyncratic factors, rather than by a true singular ‘latent’ face-processing capacity. With this in mind, we will consider the evidence relating to the *face-inversion effect* which offers further evidence that these unfamiliar face-matching tasks fail to measure such a singular ‘latent’ face-processing ability.

## Unfamiliar face matching and the inversion effect – clues to a fundamental problem

The face-inversion effect relates to the general observation that participants tend to perform more poorly on a given face task if items are presented upside-down (Farah et al., 1995; Freire et al., 2000; Yin, 1969). Generally, face-inverted performance is about 20% poorer than upright (Robbins & McKone, 2007; Rossion, 2008). Importantly, interpretations of this effect have drawn on the concept of configural processing (a key perceptual process that has implications for encoding). The concept of configural (sometimes also called ‘holistic’) face processing is that it involves the simultaneous integration of the multiple features of a face that are fused into a single perceptual representation (Rossion, 2008). Early evidence for this automatic integration of a face into a ‘whole’ singular representation emerged from work reporting the Thatcher Illusion (Thompson, 1980), in which it was shown that when presented with inverted images of otherwise familiar people, where the internal features of the face were distorted (i.e., the eyes and mouth were inverted), participants often failed to notice these distortions – despite a near trivial realisation when faces were upright. Other later work found inversion performance on the face composite illusion (e.g., Hole, 1994) – that is, this key effect is more profound with upright but not inverted faces – implying that when faces are upright, again participants cannot fail to perceive the parts independent of the ‘whole’ or its entire ‘configuration’ (Farah et al., 1998). As a consequence, the face-inversion effect (i.e., poorer inverted face performance) has been argued to ‘disrupt’ the vital configural processing need to typically recognise faces (Maurer et al., 2002); put simply, if we typically use such configural processes in recognising faces and they are subsequently disrupted, it naturally follows that performance will decline. In the same vein, researchers have thus argued that in the case of acquired prosopagnosia, a key root cause for their impairments with faces is linked to an inability to perceive faces holistically (e.g., Avidan et al., 2011; Jansari et al., 2015; Sergent & Signoret, 1992), and this is why this inversion effect may not be observed in acquired prosopagnosia (i.e., severely impaired face-recognition ability following brain injury; Farah et al., 1995).

At this point then it should be clear that a key interpretation of the inversion effect is that faces as a class of stimuli are in some manner transformed by the act of inversion (they become a ‘class apart’) – though it remains perhaps open to debate that this transformation entails presented faces that are perceived in a fundamentally *qualitatively* or *quantitatively* different manner (Rossion, 2008; Tanaka & Gordon, 2011). It is with this former interpretation in mind, that a key paper on unfamiliar face matching by Megreya and Burton (2006) is relevant here. These researchers explored the observed correlation on unfamiliar face matching and (a) inverted unfamiliar face matching, (b) upright familiar face matching and (c) inverted familiar face matching with tasks. To understand the logic for the motivation of this study, you can ask a simple question: if inverted faces are a ‘class apart’ (qualitatively different), does participant within-task performance across inverted and upright conditions correlate*?* At this point it is worth drawing the parallel with our earlier discussion of construct validity – since we established that this is typically examined through a process of both *convergent* and *discriminant* validity correlational comparisons. To reiterate, this psychometric approach assumes if two tests or conditions are appropriately tapping the same underlying ‘construct’ of a functional (cognitive) ‘latent’ process, they should be strongly associated and should not otherwise. Interestingly, the parallel here is that those advocating for the *qualitative* change theoretical interpretation of the face-inversion effect (i.e., disruption of ‘configural processing’) are also naturally making a *discriminant* validity prediction – that one should *not* observe large cross-condition correlations. On the other hand, if high correlations between inverted/upright matching *is* observed this would likely suggest – that with unfamiliar face matching at least – a similar local feature-based approach (idiosyncratic comparisons of hair, nose, eyes, etc.) is being adopted, which occurs regardless of the orientation of presentation.

Table 1 presents the observed correlations between unfamiliar face-matching accuracy for both inverted unfamiliar faces, reported by Megreya and Burton in Experiments 2–4, and upright/inverted familiar faces (Experiments 5 and 6). For unfamiliar face matching there are often high within-task correlations in performance across upright/inverted faces (Experiment 2–0.818; Experiment 3–0.730; Experiment 4–0.772); yet, unfamiliar upright face matching and familiar face- (or ‘familiarised’ face) matching performance showed small and non-significant correlations (or ‘familiarised’ face) unless these were also inverted. Moreover, there was similarly no correlation between familiar face upright/inverted conditions, consistent with other work (Valentine, 1988). The findings across a number of their experiments appear to be clear – performance with upright unfamiliar faces was consistently strongly correlated with inverted faces but not with upright familiar faces. The authors interpret these observations straightforwardly: “*We take these results to imply that unfamiliar face matching is qualitatively different from familiar face recognition”.* Namely, when faced with two images of unfamiliar faces, an observer’s best route to success is to some degree ‘image matching’, and this reflects the fact that *this constitutes the only information available* for observers to use. This situation changes if presented faces are either already familiar or become familiarised (since other long-term memory representational information comes into play) and it is these stimuli that constitute a ‘class apart’.

**Table 1:** Pearson’s correlations between accuracy performance on matching upright unfamiliar faces and other tasks across experiments (from Megreya & Burton, 2006)

Task	Accuracy
Experiment 2	
Unfamiliar faces (lineup task, target inverted)	0.818**
Experiment 3	
Unfamiliar faces (lineup task, all faces inverted)	0.730*
Experiment 4	
Unfamiliar faces (match/mismatch task, both faces inverted)	0.772*
Experiment 5	
Unfamiliar faces (upright, match from memory)	0.820*
Familiar face decision (upright, accuracy)	0.010
Familiar face decision (inverted, accuracy)	0.612*
Familiar face decision (upright RT)	−0.292
Familiar face decision (inverted RT)	−0.088
Experiment 6	
Familiarized faces (upright)	0.277
Familiarized faces (inverted)	_573**

*p < 0.05. **p < 0.01

If one assumes inverted faces *are* qualitatively different from the usual members of the face category (i.e., upright familiar faces), then these findings suggest the consequences of this inversion manipulation differ across familiar/unfamiliar faces – at least for unfamiliar face identity-matching tasks. This is the interpretation the authors draw – “*we suggest that unfamiliar faces are not processed (for identity) like faces, in exactly the same sense in which inverted faces are not processed like faces. Therefore, conflating familiar and unfamiliar faces into a single theory of face processing seems to be an unpromising approach…. Given the large processing differences between these two types of visual stimulus, development of a satisfactory account of face learning poses a significant challenge”.* The implications of this interpretation draw upon an early distinction made in the cognitive model of Bruce and Young (1986) in which they suggested a differentiation between *pictorial* and *structural* codes; with the former referring to superficial image-based information (true for unfamiliar faces be they upright or inverted), and the latter based on perceptually learnt information drawn from the process of a person’s face becoming more familiar (and sensitive to familiar face inversion). As stressed earlier, the interpretation is that when presented with unfamiliar faces an observer is more likely to be drawing on information that is quite specific (and idiosyncratic) to the image presented, and this can explain why it can be easier to recognise when one has previously seen a *particular image* of an unfamiliar face than it is for a familiar face (Armann et al., 2016). The fact that Megreya and Burton (2006) found no such correlation for familiar faces (see also Valentine, 1988) is also consistent with this overall proposal.

Megreya and Burton (2006) thus make an important point about the interpretation of testing of unfamiliar face matching that could *also* explain why others observe the poor cross-task correlations discussed earlier (see Fig. 1). In that, they suggest, performance on any given matching task has little to say about a singular ‘construct’ of ‘real-world’ face recognition ability (akin to “f”; see Verhallen et al., 2017), and instead reflects idiosyncratic factors (such as matching ‘strategies’) that are image and likely task specific (i.e., the man in these two images has similar facial hair). Changes to the presented images (as one does across face-matching tasks) and what participants specifically ‘do’, thus provokes these low-cross task-observed correlations. Concurrently, such idiosyncratic factors are largely consistently used within task regardless of the orientation format of the stimuli, and thus the *within-*task upright/inverted correlations are observed to be much higher. Returning to Bobak et al. (2023), these authors reported their highest observed correlation was for the upright and inverted versions *within* a key unfamiliar face-matching task (EFCT; Fig. 1). Put simply, all this evidence suggests unfamiliar face-matching tests have poor *construct validity* for testing hypotheses about the latent ability of individuals in many everyday familiar face-processing contexts. Our current contribution is to revisit the basic logic for the original work of Megreya and Burton (2006), with an analysis of a different data set provided by the recent work of Bell et al., (2023). In their case the focus was largely on a dissociation of performance across identity and emotion matching tasks for two key samples – 133 controls versus 124 DPs. However, to their credit the authors included a variety of face-matching paradigms (discussed below) and included upright/inverted presentation conditions – this latter element clearly being useful to reconsider the work of Megreya and Burton (2006). Thus, in our case, we will only focus on the control sample, to examine both (a) *within-task* correlations for upright/inverted conditions (akin to Megreya & Burton, 2006) and (b) *across-task* correlations (akin to Bobak et al., 2023) within subjects. Given the commendably thorough nature of their work, we will also have two other interesting additions: (1) we can provide analyses for both accuracy and reaction time (RT) measures (as yet unexplored) and (2) we will investigate these correlations across identity matching and emotion expression matching judgement tasks. Megreya and Burton (2006) pointed out that their conclusions around unfamiliar faces related to *identity* only. In other words, the authors interpreted both the observation of (and consequences following) large upright/inverted within-task correlations as being largely limited to unfamiliar face identity-matching tasks (such as those considered by Bobak and colleagues). Other face-judgement tasks, such as emotional expression, are argued not to provoke similar high upright/inverted correlations– and there is early evidence in support of this position. For example, Zhou and Jenkins (2020) ran a within participant study using identity matching, gaze direction and emotion judgements and found no correlations between tasks – other studies have reported similar results using between-groups designs (see, e.g., Duchaine et al., 2009; Young et al., 1993). Consequently, the current work can achieve two objectives simultaneously – firstly, to determine whether they key findings of Megreya and Burton (2006) are ubiquitous to *all* kinds of unfamiliar upright/inverted face-matching tasks across accuracy/RTs, and secondly whether similar high within-task correlations are *also* observed for emotion upright/inverted expression matching tasks.

## Methods

Our data analysis involved the responses of a sample of 133 controls tested on a variety of unfamiliar face-processing tasks. These tasks are described in detail in the original work of Bell, Duchaine, and Susio (2023), and we would advise readers to turn their attention to this paper for specific details.^1^ Nonetheless, these tasks fall into two fundamentally different types – namely, *identity* matching in which participants were asked to select items on the basis of whether the pictures represented the same unfamiliar individual, and *emotion* matching in which participants had to judge if pictures of unfamiliar faces represented the same emotion (i.e., happy, angry, disgust, fear and surprise). The format of matching in each case comprised either (a) *simultaneous* presentation, in which all faces are presented on the screen at once and the participant makes their judgement, (b) *sequential* presentation, in which participants saw two faces one after the other and made their judgement, and (c) a *sorting* task, in which participants were asked to sort six faces with regard to their similarity (in emotion or identity) with a target face, with all information remaining on the screen at the same time. Importantly for our purposes, to reiterate, in all these cases for half the trials the unfamiliar faces were presented upright and half inverted. Bell and colleagues collected both accuracy and RT data. In our analyses, we initially present the straightforward correlations between all task conditions for each measure type to examine their general patterns (akin to the table presented above) for both of our two dependent variables. We then undertake two exploratory factor analyses (EFAs) on the same two datasets (accuracy and RT) to uncover the latent variables, if any, that might give rise to the pattern of correlations we observed – and critically will determine if such variables reveal *converge* or *discriminant* for performance on the upright and inverted conditions. This psychometric perspective allows for the discovery of common factors that may represent distinct or shared psychological constructs that relate to responses on these tasks.

## Results

### Correlation analysis for accuracy across sorting, sequential and simultaneous face-matching tasks

**Fig. 2:**
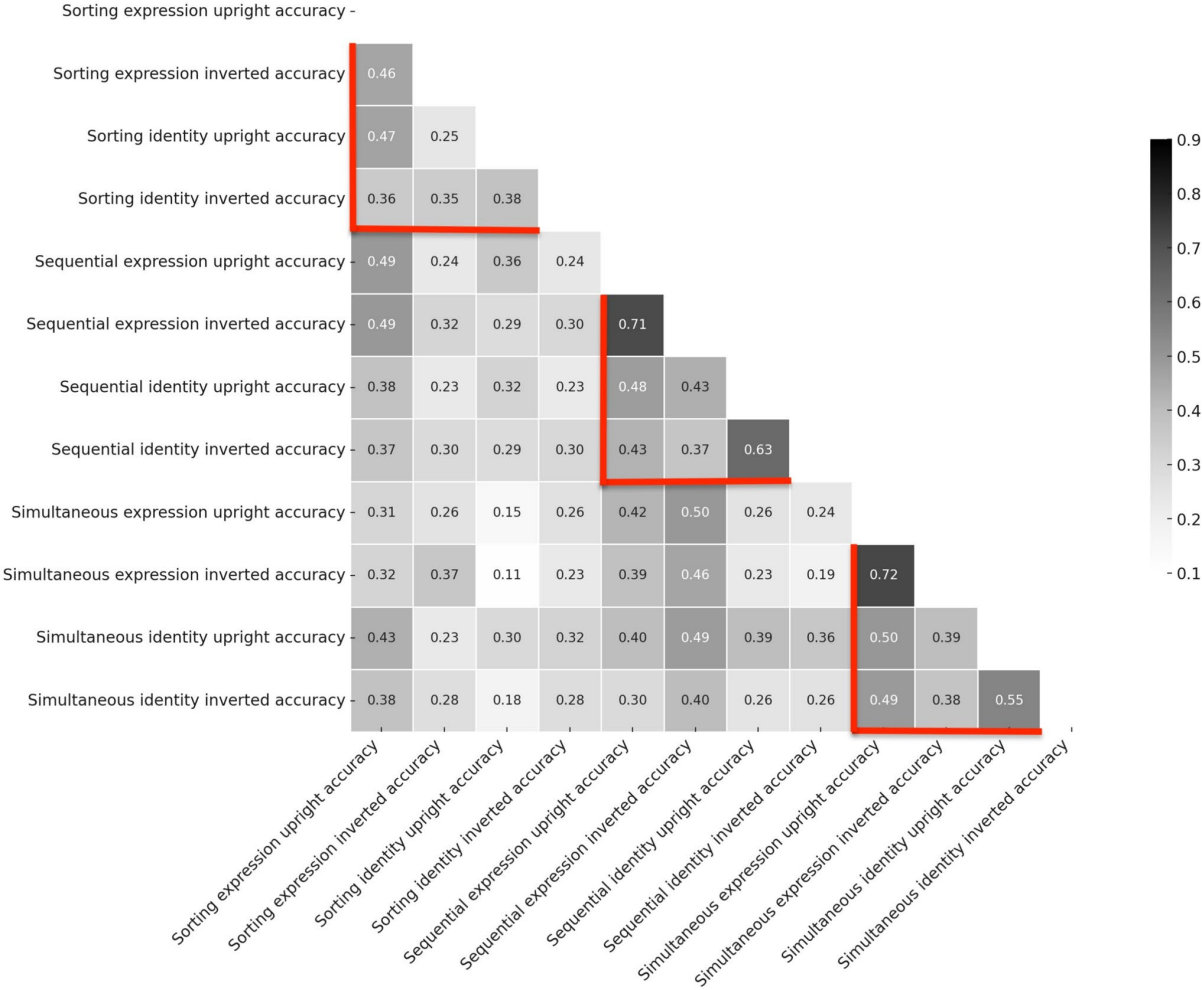
Correlation matrix for accuracy scores across all task conditions

We first produce correlation matrices (or ‘heatmaps’), illustrating all within and cross-task associations, shown in Fig. 2. Bell and colleagues tested two different kinds of judgement from faces (identity and expression), two different kinds of presentation (upright/inverted), and three different *types* of tasks: (a) sorting tasks (b) sequential tasks, and (c) simultaneous tasks. In Fig. 2 the different correlations relating to all conditions *within* these three paradigm types are highlighted by red borders: at the top left are all correlations for the conditions within the sorting task, in the middle quadrant are all those for within the sequential task, and in the bottom are those for within the simultaneous task. These correlations also vary in magnitude, being somewhat lower for the sorting task conditions, with effect sizes ranging from small to large (Cohen, 1988): sorting task (range 0.25–0.47), sequential task (range 0.37–0.71), and simultaneous task (range 0.38–0.72), Critically these within-task type correlations are observed *regardless* of type of face judgement (i.e., identity/expression) or presentation format (i.e., upright/inverted). In addition, many correlations are even observed *across* task types (i.e.., emotion/identity judgement), albeit to a smaller general magnitude. We provide more detail for all these analyses in Appendix Table 2 along with upper and lower confidence intervals and statistical significance (with uncorrected, Holm and Benjami-Hochberg False Discovery Rate (FDR) p-value corrections). In sum, the matrix presented in Fig. 2 provides a global impression of the correlations for accuracy across all task conditions and suggests a pattern in which almost everything correlates with everything else to some degree.

**Fig. 3:**
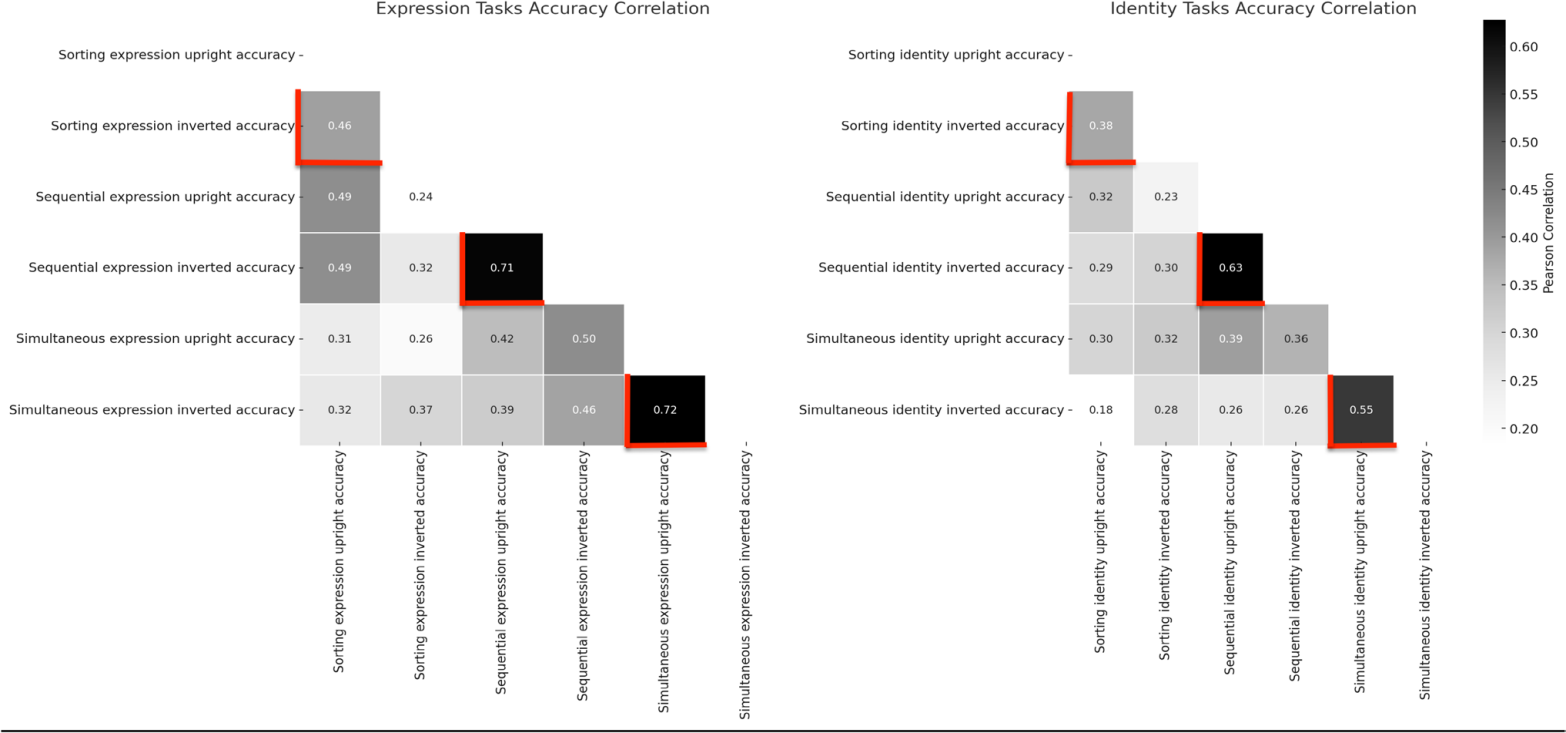
Correlation matrix for accuracy in identity and emotion judgement tasks

However, given our work is inspired by that of Megreya and Burton (2006), we will turn our attention to the key correlations across upright/inverted conditions for within-task identity matching, whilst also considering the same for emotion matching. In Fig. 3 we present this information side by side. In this case, the key observation is that the *within-*task upright/inverted condition correlations for each of the task types (marked with red borders) appear of equivalent magnitude regardless of *decision type* – sorting task (identity 0.38, expression 0.46), sequential task (identity 0.63, expression 0.71), simultaneous task (identity 0.55, expression 0.72). The correlations are clearly higher for the sequential/simultaneous presentations, but there is no evidence that this relative difference pattern varies across judgement types. Namely, it appears that the observed correlations within given tasks for the upright/inverted conditions do not generally differ much in magnitude if one is asking participants to make matching judgements of emotional expression or identity. The pattern of correlations suggests that in general, upright and inverted unfamiliar face matching is (a) most strongly associated within tasks (to levels akin to Megreya & Burton, 2006 and Bobak et al., 2023) regardless of paradigm type, (b) observed to a similar degree for *both* identity and emotion expression judgement within tasks, and (c) even observed (albeit to a lower degree) *across* different task conditions.

### Correlation analysis for reaction times across sorting, sequential and simultaneous face-matching tasks

**Fig. 4:**
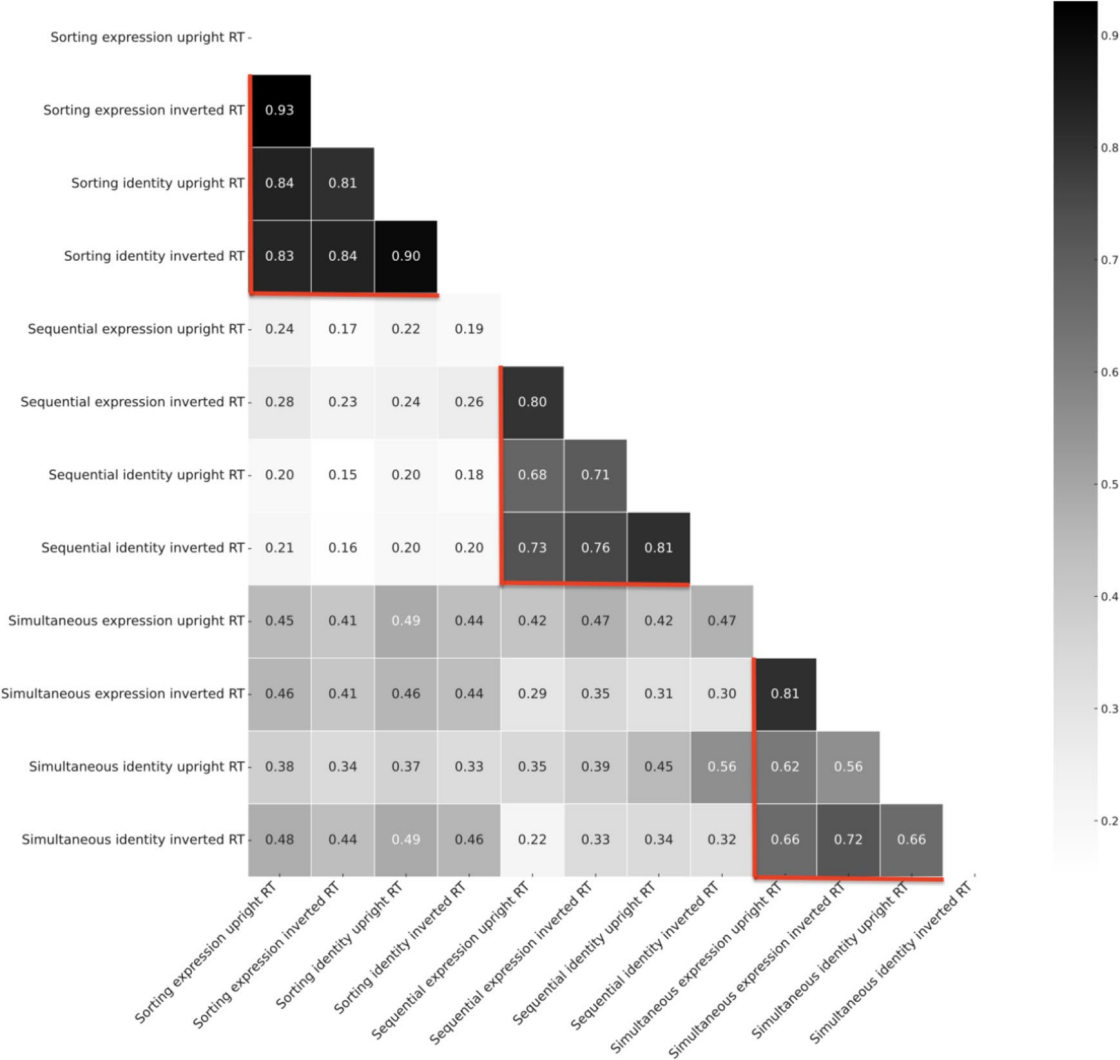
Correlation matrix for reaction times (RTs) across all task conditions

**Fig. 5:**
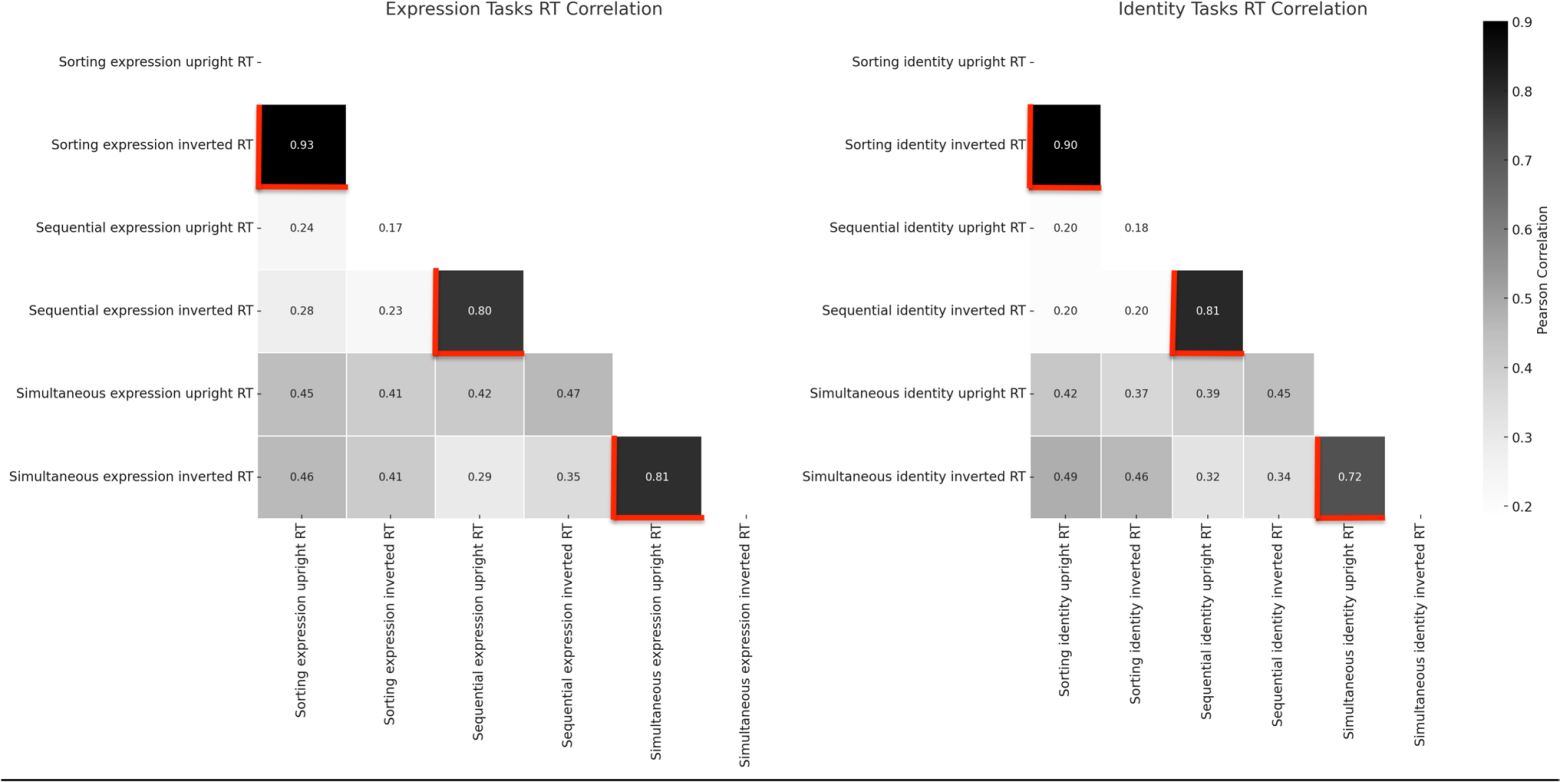
Correlation matrix for reaction times (RTs) in identity and emotion judgement tasks

As before, the key correlations are presented in a heatmap for the three task types for both identity and expression judgements and presentation type (see Fig. 4 below). As with the accuracy picture discussed earlier, there is clear evidence of strong *within-*task correlations relating to these three paradigm types (highlighted by the red borders marked in Fig. 4). In fact, in this case these correlations appear to present a much clearer high correlation story than that for accuracy – Sorting tasks (range from 0.81–0.93), Sequential tasks (range from 0.68–0.81), and Simultaneous tasks (range from 0.56–0.81). In addition, and as earlier observed, there are also examples of cross-task correlations. For further detail on all these analyses please refer to Appendix Table 3. Overall, Fig. 4 provides a global impression of a set of widely correlated variables, whichever combination of tasks are considered. Finally, we again focus specifically on the identity and emotion judgements – and present these correlation matrices in Fig. 5 side by side. As before, the purpose here is to focus on *within-*task upright/inverted conditions (marked with red borders), to provide some comparison with the observations of Megreya and Burton (2006). In the case of the RT dependent variable, we can observe high within task correlations in all cases, with no real evidence of differences in magnitudes across task types (unlike accuracy) or judgement types (i.e., emotion/identity). For context, these correlations are of a similar in magnitude to those reported by Megreya and Burton (Experiment 2–0.818, Experiment 3–0.730, Experiment 4–0.772) for reported accuracy. On balance we have clearly replicated the observations of Megreya and Burton (2006), but in a much more *extreme* manner. It seems upright/inverted unfamiliar face-matching performance is correlated within task, regardless of the kind of paradigm (i.e., simultaneous, sequential or sorting tasks) or even the kind of decision (identity or emotion judgement).

### Exploratory factor analysis for accuracy and reaction time

Beyond exploring the correlation structure of the tasks, we separately subjected both aspects of the dataset (accuracy and RT) to an EFA to identify the underlying latent variables that give rise to the common variance amongst the 12 observed variables. These latent variables may speak to a distinct but correlated process, or a unified process, that underpins responses on these tasks.

#### Accuracy EFA

**Fig. 6:**
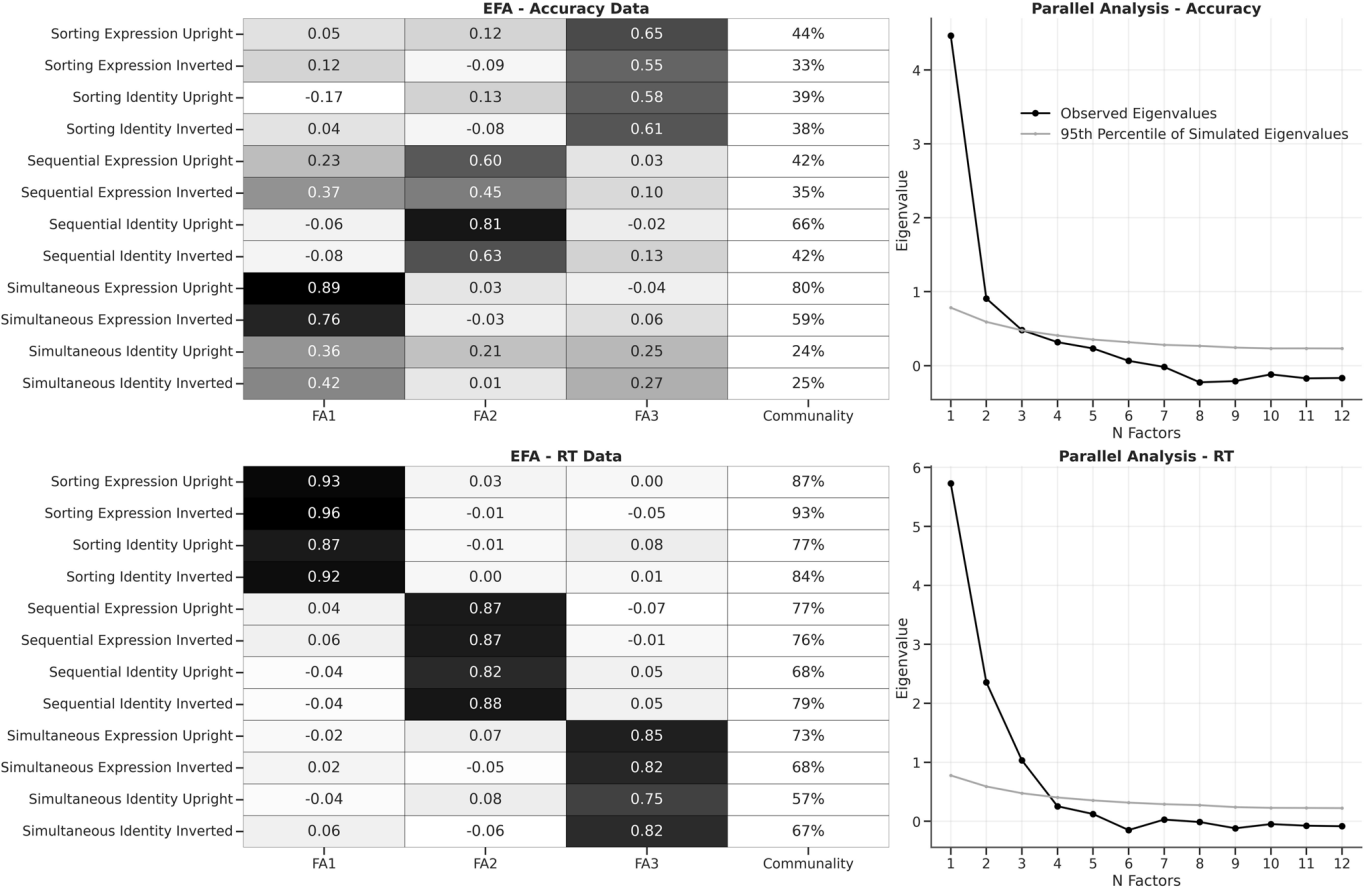
Left column – loading matrices and communalities of both accuracy and reaction time (RT) exploratory factor analysis (EFA). Right column – results of parallel analysis showing the observed eigenvalues and 95 th percentile of simulated eigenvalues

The first EFA was conducted using the 12 accuracy scores across the different task conditions. Unsurprisingly, Bartlett’s sphericity test was significant, χ^2^(66) = 631.92, *p* < 0.001, indicating the accuracy data correlation matrix differed from an identity matrix, and the average Kaiser-Myer-Olkin (KMO) score was 0.85, suggested a large degree of common variance. We selected the number of factors using parallel analysis, simulating 5,000 datasets with uncorrelated variables of the same size and shape as the accuracy data and computed the factor-analytic corrected eigenvalues of these datasets (Dinno, 2014). We retained the number of factors for which the observed eigenvalues of the accuracy data were strictly greater than the 95 th percentile of the simulated eigenvalues (this corresponds to the line presented in the parallel analysis plot). This suggested a three-factor solution, which we extracted with a minimum-residuals EFA with oblimin rotation. Factor loadings are shown in Fig. 6 (top row) showing the extracted factors essentially represent the different types of task presentation (sorting, sequential, and simultaneous), with only a few tasks sharing similar loading strengths with other factors (e.g., simultaneous inverted expression). Together, the factors explained 44% of the variance in the data (16%, 14%, and 13% for factors 1, 2 and 3, respectively). The communalities (how much variance the factors explained in the variables themselves) varied greatly from 24 to 80%.

Consequently, we appear to have identified three factors that each specifically underpins latent ability accuracy *within* each of the key experimental paradigms (i.e., sequential/simultaneous presentation and sorting) regardless of the orientation of stimulus presentation or decision type. This provides important context for the interpretation of the correlation matrices presented in Fig. 3.

#### RT EFA

The second EFA was conducted using the 12 RT scores across the different task conditions. A Bartlett’s sphericity test was also significant, χ^2^(66) = 1477.03, *p* < 0.001, with a similarly high KMO score of 0.84. Parallel analysis similarly suggested three factors, extracted with the same minimum residual and oblimin rotation specification (see Fig. 6 bottom half). Again, the factor loadings clearly represent the different types of task presentation as opposed to correlations amongst orientation or expression version identity. These factors explained 75% of the variance in the data (28%, 25%, and 22%). The communalities were quite high, ranging from 57 to 93%. Overall, our analysis of RTs aligns with the earlier accuracy analysis; suggesting three distinct factors are linked to *task specific* ‘latent’ abilities relating to each of the key experimental paradigms: sequential presentation, simultaneous presentation, and sorting. Notably, there is no evidence that the performance *within* a given task for upright/inverted conditions or the type of decision (identity or expression) diverge across this observation. Instead, this suggests that differences in face-matching speed are primarily tied to the *specific paradigm* being used. This conclusion is consistent with the pattern across correlation matrices discussed in the earlier figures, emphasizing that performance within a specific task is the most relevant factor. What is particularly interesting here is that these factor analyses constitute the first of their type in that they consider accuracy and RT performance *across and within* different kinds of unfamiliar face-matching tasks, where we combine conditions that have previously been assumed to be linked to either convergent validity (i.e., performance *across* all the upright identity-matching task conditions as a ‘latent’ ability) or assumed to be linked to discriminant validity (i.e., across identity/emotion expression judgements and upright/inverted presentations *within tasks*) – and yet we observe little evidence for either of these predicted patterns.

## General discussion

The findings of the current work can be summed up succinctly by the general observation that unfamiliar face-matching task conditions appear to largely correlate to some degree within tasks across the board – regardless of task type, judgement type or format of orientation, with the general correlation size being larger for RTs than accuracy. EFA equally suggests no evidence of orientation differentiation and confirms that performance is generally *task specific* with respect to ‘latent variables’. From the perspective of the principles of test validation and the interpretation of correlation magnitude, we have already mentioned the psychometric concepts of convergent and discriminant validity (see *Introduction*). With that in mind, it seems fair to say that the conditions that should be demonstrating discriminant validity (i.e., inverted conditions) do not since observed correlations are too high. Whilst the conditions that should be demonstrating convergent validity for a single ‘latent construct’ of ‘every day’ face processing (i.e., upright faces across different test types) do not since observed correlations are quite low (see Figs. 3 and 5). That is, with respect to the typical psychometric goals in this context, the expected pattern is generally *inverted*! It is therefore worth reflecting on these different correlational observations separately – namely, how best to interpret the consistently high observed correlations we see within tasks for matching upright/inverted faces, which is consistent with the key work of Megreya and Burton (2006). While at the same time reflecting on the much lower correlations observed *across* face-matching tasks for upright faces and thus lack of overlapping latent construct, which mirrors the findings of other key work by Bobak and colleagues (see also Fysh et al., 2020), who similarly reported low correlations *across* upright face processing tasks. We will discuss each of these separately.

### Matching upright and inverted unfamiliar faces – interpreting a persistently high within task correlation

Our work was largely inspired by Megreya and Burton (2006), who observed that across a series of studies, upright and inverted unfamiliar face matching performance was often highly correlated (i.e., for Experiments 2–4 ranging from 0.730 to 0.818, see Table 1). In our case we observed similarly high within-task upright/inverted correlations regardless of the type of paradigm (sequential, simultaneous, sorting) or the dependent variable (i.e., accuracy/RT) and regardless of the kind of judgement (i.e., identity or emotional expression). In addition, there was little evidence from the EFA analyses that within task upright/inverted condition performance was non-overlapping either, rather it suggests that the specific types of tasks were linked to separate ‘latent abilities’, consistent with the likely use of task specific *idiosyncratic* image specific heuristics being employed by participants in each case. That is, participants are doing *task- and trial-specific things*, on each of these different face-matching tasks (Dunn et al., 2024). We thus clearly replicate the observations of Megreya and Burton (2006) and extend them substantially. Importantly we found, with unfamiliar face matching performance at least, no evidence for a singular construct relating to a kind of ‘general face-perception factor’ (akin to ‘*f*’; Verhallen et al., 2017).

Therefore, we are left with the question of how to interpret this observation of high correlations *within* tasks for upright/inverted unfamiliar face matching. Such an interpretation will rest on the extent to which one sees inverted face stimuli as either quantitatively different from upright faces (i.e., they are just much harder items) or qualitatively different (i.e., they are not seen as ‘faces’ at all). As we established in the *Introduction*, this latter interpretation is favoured by the work of Megreya and Burton (2006) – to remind the reader, they argue that such high correlations imply that unfamiliar face-matching tasks likely do not involve conventional (familiar) face-processing systems. Rather, for such tasks participants employ quite piecemeal (idiosyncratic) comparison strategies (e.g., hair lines, shape of nose etc.) – and to foreshadow the next section, these ‘local’ image matching strategies would naturally not be expected to generalise across tasks. Interestingly, they reserved this interpretation for unfamiliar face matching on identity, but the current work clearly demonstrates this extends across unfamiliar face matching for *both* identity and emotion. Finally, if one assumes that the matching of upright unfamiliar faces reflects a single ‘latent’ ability likely related to the ‘construct’ of general (‘everyday’) face processing, it is apparent that validity is poor. Consequently, it is also important to consider the observed cross-task correlations, particularly for upright familiar face judgements, since they naturally cannot be entirely explained by such ‘local’ task specific elements.

### How do we interpret the consistently low correlations across face-matching tasks?

Independent of the issue relating to upright/inverted presentation within tasks, is the consistently reported pattern of lower correlations across unfamiliar upright face-matching tasks (see Fig. 1), despite being very similar in many respects. As ever, low correlations remain a challenge to interpret, since classical ‘statistical significance’ is not always helpful. One option is that if one assumes all unfamiliar face-matching tasks comprise some converging *feature matching* process, perhaps the lower cross-task correlations reflect this process in action? However, it is also very plausible that multiple conditions across tasks will be correlated simply because of factors like generic task performance components linked to low-level perceptual/motor response elements or perhaps non-perceptual factors specific to participants such as motivation or personality traits (e.g., conscientiousness). That is, it remains possible that aspects of any observed correlations will have very little to do with the processing-specific (cognitive) component of interest (such as ‘featural processing’ generally).

This motivates the approach discussed earlier about using measures that can establish *both* convergent (high) and discriminant (low) observed correlations during test validation – to quote Wilmer et al., (2012a, b) “*the best evidence for discriminant validity comes from a low correlation [r* = *0.1–02] with a test designed to capture a similar process or domain* *(a “proximal dissociation”).* It is therefore apparent that many cross-task correlations we have observed are often low (or near low) enough to meet the criteria for this discriminant validity outcome. We can further demonstrate this point with two key examples. Firstly, the historical example provided by the work of Weschler during the development of his clinical assessment battery known as the Weschler Memory Scale (Weschler, 1997). In this case, the principal objective was to create a sub-set of tests that constituted a ‘general measure of visual memory’, and it was observed that the face test in the battery had a ‘poor’ correlation with the other included visual memory tests (r = 0.28 to 0.30; see Holdnack & Dellis, 2004, Millis et al., 1999). Unsurprisingly, these correlations were interpreted by Weschler as *too low* for his goal (convergent validity of ‘general’ memory construct), and thus face materials were removed from the test completely in subsequent development of the WMS-IV (see Wilmer et al., 2014, and Wilmer et al., 2010, for a discussion). A second, concrete example is provided by Wilmer et al., (2012a, 2012b), who validated the Cambridge Face memory Test (CFMT) using the convergent/discriminant approach; in the latter case *discriminant validity* was demonstrated by correlating the CFMT with a non-face task (the Abstract Art Memory test; Wilmer et al., 2010), for which the corresponding interpreted ‘*low*’ correlation (*r* = *0.26*) was deemed appropriate. These two examples essentially provide some context of what level psychometricians would consider a ‘*low correlation*’ for the purposes of demonstrating discriminant validity.

However, at this point many experimental psychologists may wish to point out the fact that such a level of observed correlation is still likely to be ‘statistically significant’ (particularly with large Ns) and thus be reluctant to dismiss it – since many theoretical interpretations can often hinge on such small effect sizes. It is worth taking a small historical diversion into why some psychometricians are inclined to see such ‘low non-zero correlations’ (e.g., ranging from 0.10 to 0.30) as inconsequential. In psychometrics, there has been considerable discussion around the observation that in much behavioural correlational research, *non-zero* correlations appear omnipresent in *any* large multivariate data sample, with some quite serious implications (Meehl, 1990). If non-zero correlations are always observed, one might conclude that the null hypothesis (i.e., of no association whatsoever) is *always* false. Unfortunately, this will have consequentially severe implications for the interpretation of ‘statistical significance’ under the conventions of null hypothesis significance testing (NHST), since power analysis demonstrates that with a sufficiently large sample size (e.g., 400 +), small effect sizes will often meet ‘statistical significance’. In which case with sufficient ‘power’, a pretty theoretical tale can be legitimately told under such conventions of statistical interpretation. This has important empirical consequences for *all* researchers interpreting correlations as a means of undertaking experimental work – and has led to the extended discussion of the concept of a ‘*crud factor*’ (e.g., Meehl, 1986; Orben & Lakens, 2020)*.* This is the epistemological concept that, in correlational research, all variables are connected through causal structures, which will frequently result in real non-zero correlations between *all* variables in any given data set – that is, *everything (non-zero) correlates everywhere all at once* (the inspiration for our paper’s title). Naturally, the debate has warranted discussion of what constitutes the size of this ‘crud’, and *crud estimates* vary (e.g., Meehl, 1990 has an estimate of r = 0.3). In any case, as any correlation approaches a smaller and smaller effect size, the certainty of its interpretation as ‘crud’ or otherwise becomes harder (since statistical ‘significance’ under the principles of NHST provides little help in this respect) – and it remains an open challenge to determine whether there is a meaningful ‘threshold’ of ambient association (a ‘crud estimate’) that could be used either as criteria for the dismissal of non-meaningful correlations, or even as a means for ‘correcting’ for this background noise (Orben & Lakens, 2020). Others such as McKelvie (1994) have argued that perhaps an option is to always interpret ‘crud level’ observed correlations as ‘meaningful’ if they were ‘*theoretically predicted*’ and treated as inconsequential otherwise, but this appears quite an entirely circular option to take in our view (and of course provides the opportunity for all kinds of rampant statistical interpretational ‘abuse’). For the current purposes, the above discussion provides an important context for interpreting the low correlations *across* tasks we have observed on accuracy (see Figs. 2 and 4; many of which are ‘statistically significant’; see Appendices), since it remains unclear how much this may or may not be theoretically uninteresting ‘crud’ (this also applies to Fig. 1 and the work of Bobak et al., 2023). We would advise academics in our field to bear this in mind in the future (see also Mõttus, 2022, for related discussions about the implications of low correlations from the perspective of interpreting *individual level* performance).

In sum then, many of the key *cross-task* correlations we have observed are ‘low’ in psychometric terms and thus clearly insufficient for the purposes of convergent validity – that is, we would interpret the evidence they are tapping the same singular underlying functional face processing system (latent ability) as poor. How to interpret these low correlations? Previous researchers have tended to attribute these to the fact that the different unfamiliar face-matching tasks provoke task/image specific (idiosyncratic) strategies from participants (like hair-line matching), which naturally differ across tasks and thus poor correlations follow. This interpretation is also consistent with the fact that *within task* observed correlations are consistently higher (see Figs. 3 and 5). That is whatever strategy you might employ for a specific task is true regardless of whether faces are upright/inverted or whether you are making an identity/emotion judgement. Psychometrics also provides sobering evidence that at least part of these low correlations may also just reflect the kind of ‘ambient’ association that is observed across many behaviourally similar tests and likely reflect mundane general factors such as low-level motor/perceptual processing overlap.

### If unfamiliar face-matching tasks do have poor construct validity for the purposes of measuring hypotheses about much everyday familiar face processing – what are the implications?

Overall then we appear to have two bits of evidence both of which are inconsistent with the principle that unfamiliar face-matching tasks are indeed tapping some form of singular ‘latent’ face processing ‘construct’ for general face-processing ability – on the one hand upright unfamiliar face-matching task appear to have ‘low’ correlations (see also Bobak and colleagues, and also Fysh et al., 2020, who similarly report low cross task correlations) and on the other hand high within task correlations are high across upright/inverted conditions. What implications might this have for their utility in cognitive experimental psychology that seeks to understand how we recognise those we do ‘every day’ (i.e., ‘real-world’ face processing)? We highlight several key examples in this section.

Firstly, poor construct validity has obvious implications for training designed to improve performance relating to ‘everyday’ face recognition ability, since at best such training using unfamiliar face matching will largely *only* improve ability in very specific ways. This is consistent with previous work in the context of training for border guards that involves the regular decision being made about whether the unfamiliar face of a person standing before them matches that of the image presented to them in a passport. What is interesting in this case is that training largely involves specifically focusing on particular details (i.e., features) to identify key overlaps/differences (Towler et al., 2019) – and although there is some evidence training may have utility for this specific purpose, there is little evidence of generalisation to improvements in much broader ‘real-world’ face recognition ability (White, Towler & Kemp, 2021). Specifically, this quite detailed feature matching is often the kind of slow and laborious behaviour observed by acquired prosopagnosics doing face-matching tasks (Susilo, et al., 2015), so it is perhaps ironic that a tactic of last resort used by people with acquired brain injury is the training method being advocated. Interestingly, AI software follows the same principles, and thus it’s likely that despite their current astoundingly good accuracy, whatever this software is doing has little to do with the kind of human face recognition ability cognitive psychologists are interested in – in other words, AI software functions like a super-fast acquired prosopagnosic! (Kramer, 2025). In summary, while training with unfamiliar face matching may be useful in certain contexts, the findings from this study suggest that it is unlikely to lead to broad improvements in general face-processing abilities for populations such as those with developmental prosopagnosia (for a more in-depth discussion, see Bate & Bennetts, 2014; Degutis et al., 2014; Davies-Thompson et al., 2025). An anonymous reviewer correctly noted that it remains unclear whether the limited impact observed is because face-identity processing is largely inherited (e.g., Tree et al., 2017; Wilmer, 2017) and therefore resistant to training. If so, then all training approaches are likely to achieve only modest generalisation if any.

Secondly, poor construct validity in this context for unfamiliar face-matching tasks also has important consequences for researchers who use such tests as a ‘proxy’ for the ‘construct’ of general face-processing ability in the service of asking other research questions. For example, there has been a long-standing discussion about the degree to which ‘faces are special’ (i.e., a class apart from other complex visual stimuli), and a straightforward approach to test this issue is to determine the degree to which performance on a non-face task may (or may not) be correlated with a face task (see Mahon, 2020; Towler & Tree, 2018). Clearly, we would caution the use of unfamiliar face-matching tasks in this context, simply because it may well be problematic to interpret what a high/low correlation implies (e.g., Zhou & Jenkins, 2020, discussed below). Moreover, as we have also discussed, observed correlations may be too ‘low’ in psychometric terms to meaningfully interpret in any case, despite apparent ‘statistical significance’ (see above discussion on ‘*crud*’). For example, it has been reported that word/face-processing tasks can have observed correlations (e.g., Burns & Bukach, 2022) that are statistically ‘significant’ (with sufficient power), but of magnitudes in a range (r = 0.2–0.3) that psychometricians would consider evidence for *discriminant* rather than the sought for convergent validity*.* In other words, it is very possible cross task correlations sufficient to be statistically ‘significant’ *might* imply overlapping cognitive functional processes or some other meaningful theoretically interesting observation – or they may just be artifacts of quite task/stimulus specific strategies or other more ‘mundane’ ambient associations – unfortunately the observation of the ‘low’ correlation *alone* provides no obvious answer (but see Burns et al., 2017).

Thirdly, other experimental psychologists may have the reverse objective. Many, for instance, argue that emotion and identity processing are governed by separate functional ‘systems’ (Bruce & Young, 1986), such that one may expect to see ‘low’ cross-task correlations (e.g., emotion vs. identity) consistent with *discriminant* validity. For example, Zhou and Jenkins (2020) found a low correlation (r = 0.15) between identity and expression simultaneous matching tasks, which could be interpreted as evidence for such a dissociation. However, these ‘low’ correlations might reflect limitations of particular unfamiliar face-matching tasks as proxies for the ‘constructs’ under study (see Bobak et al., 2023; Fysh et al., 2020). In other words, the low correlations might not necessarily indicate genuine discriminant validity. Instead, they may arise from image-specific (idiosyncratic) matching strategies tied to the design of the task (simultaneous vs. sequential vs. sorting). To be clear, we are not dismissing the possibility that emotion and identity judgments may indeed dissociate – especially for ‘extreme’ performers (see Bell et al., 2023). Rather, we urge caution in interpreting results from unfamiliar face tasks in drawing parallels to either non-face tasks or different judgements about faces illustrated by the previous examples. That is, different unfamiliar face-matching tasks should not be just assumed to converge on a singular ‘construct’ of underlying latent ability – and when that assumption is clearly challenged, this has a number of subsequent consequences to the ‘*derivational chain’* between test and theory that underpins our subsequent hypothesis testing assumptions (Scheel et al., 2021; Meehl., 1967; Mirman et al., 2024), given the potential issues with construct validity.

Fourthly, as was mentioned at the start of the *Introduction*, in some cases the development of unfamiliar face-matching tasks has been motivated by the goal of exploring individual differences in face processing; that is to achieve the aim that they can be used to ‘diagnose’ extremely good or poor ‘every day’ face processing performance. Clearly, if convergent validity for unfamiliar face-matching tasks is as low as appears, the utility of such tests for this purpose is likely to be poor, since ‘good’ (intact) performance may not belie ‘normal everyday’ face-processing ability. Moreover, this may also explain why such tasks tend to correlate poorly with individual differences in subjective reports of day-to-day face processing (e.g., Burns et al., 2023). As we mentioned earlier, if behaviour on simple unfamiliar face-matching tasks reflects individuals adopting various ad hoc ‘strategies’ or heuristics to achieve particular task success, then interpreting test performance with respect to the ‘construct’ of latent abilities for day-to-day ‘real-world’ face-processing ability is likely flawed. Although we wish to be clear this does not necessarily imply unfamiliar face tasks have *no* utility – it is clear they are very likely related to the ‘construct’ of the experience of passport controllers (and similar contexts) that we have highlighted earlier in our discussion of training. In fact, they likely also have utility in such specific applied contexts where the recruitment of high performers to undertake such specific tasks is the goal (e.g., *BeSure®*; Ramon & Rjosk, 2022; Ramon, 2021).

In any case, the solution to measures that have better construct validity with respect to ‘everyday’ latent face abilities must lie in the development of new tests that better match ‘real-world’ scenarios and thus are tightly aligned with the appropriate underlying *construct*. In particular, the investigation of variability in ‘everyday’ individual differences could more profitably focus on familiar faces or paradigms that explore the consequences of *familiarisation* to faces (i.e., learning paradigms; see Popova & Wiese, 2023). Moreover, it is also possible that our conclusions about unfamiliar faces reflect the use of photographs as the primary stimulus, it is possible the use of more ‘ecologically valid’ stimuli (e.g., colour and/or moving images – see Longmore & Tree, 2013, or even digital avatars with faces of real people – see Fysh et al., 2022) might change things. At the same time, we emphasize that manipulating face orientation generally impairs face-processing ability, regardless of familiarity. It is therefore crucial to distinguish between performance changes due to the possible general disruption of ‘configural’ processing and those related to familiarization processes. Although inversion reduces performance for both familiar and unfamiliar faces, it remains unclear whether this represents a *uniform* quantitative shift or qualitatively *distinct* mechanisms (Megreya & Burton, 2006). Our findings suggest that these differences have often been conflated – particularly in interpreting inversion superiority effects with unfamiliar faces versus the extremely poor upright performance seen in prosopagnosia. The challenge lies in recognizing that face-matching tasks may tap into fundamentally different processes for familiar and unfamiliar faces. Megreya and Burton (2006) argued that unfamiliar faces are, in effect, *“not faces”* in this context. Building on their work, we propose two additional key points.

Firstly, this proposal hinges not just on the observed high correlations for within task upright/inverted unfamiliar faces we have observed, but *also* on low observed correlations for the same with respect to familiar faces (Megreya & Burton, 2006) – a classic convergent/discriminant validity pattern discussed earlier. However, we would urge further investigation of the latter pattern in future. Given the apparent overlap between upright/inverted unfamiliar face matching performance for both identity and emotion judgements tasks, it may be of interest to determine if this correlation is attenuated for face-emotion judgements when faces are familiar (or familiarised) in a similar manner to that observed by Megreya and Burton (2006) for identity judgements (discussed earlier). Since the evidence, so far, of low upright/inverted correlations is largely limited to matching of identity with familiar faces – we would argue the general pattern of familiar(ised) faces and observed upright/inverted correlations could be explored further in more general terms. In addition, it is worthwhile reporting correlations for upright/inverted matching tasks across stimulus class, such as with cars, houses, words, etc., with a similarly large within participant data set and undertake a similar EFA approach to that used here. Though we would also stress *very* careful test construction is needed here and would advocate the utility of item response theory approaches for this objective (see Brysbaert, 2024).

Secondly, if we are inclined to accept the interpretation that familiar/unfamiliar faces reflect *qualitatively* different types of stimuli, we would suggest that it is *not* unfamiliar faces that are ‘not faces’ (Megreya & Burton, 2006), but actually the reverse! If one defines the word ‘face’ as purely a generic member of a particular visual stimulus class, in the same way as a ‘dog’ or a ‘table’ might be, then it is in fact, unfamiliar faces that are the best examples of this type. This is *not* the case for highly familiar faces, since these are instances of *unique exemplars* of this visual category – they constitute *so much more* than being a face – in fact it has long been pointed out that what makes faces ‘special’ is instances when they represent specific familiar people (*my* father, *my* friend), and thus the appropriate comparison performance would be testing with like kind (*my* car, *my* book – see Damasio et al., 1982). Curiously, this is rarely, if ever, the key comparison explored when trying to resolve the ‘are faces special’ debate (see the classic McNeil & Warrington, 1993, for an exception).

In sum, we would argue that the issue of test construct validity warrants much closer attention by researchers in the field, which echoes the sentiments of Fried and Flake (2018) when they write *“If a scale lacks validity or measures different constructs across samples, there is little benefit in conducting replication studies. We must take a step back and discern how to define and measure the variables of interest in the first place. In such cases, what we need are validity studies, not replication studies”.* We hope the current work, can spur this call to action and we can ensure the measures used have appropriate construct validity in the future. Psychometrics as a field provides some excellent lessons to guide test development, and yet experimentalists have historically tended to ignore their guidance, perhaps if nothing else our work suggests this should no longer continue (see Brysbaert, 2024, for an excellent primer on these approaches).

## Data Availability

The publication undertakes secondary analysis on datasets provided by a previous publication and are freely available (Bell et al., 2023: Dissociations between face identity and face expression processing in developmental prosopagnosia. *Cognition*, *238*, 105,469).

## Appendix

**Table 2:** accuracy correlations between different conditions

Test 1	Test 2	Pearson’s r	Lower 95% CI	Upper 95% CI	p-value (uncorrected)	Holm p-value	Benjamini–Hochberg p-value
Sorting expression upright accuracy	Sorting expression inverted accuracy	0.462	0.317	0.586	< 0.001	0.0066	0.0001
Sorting expression upright accuracy	Sequential expression upright accuracy	0.493	0.353	0.612	< 0.001	0.0066	0.0001
Sorting expression upright accuracy	Sequential expression inverted accuracy	0.494	0.354	0.613	< 0.001	0.0066	0.0001
Sorting expression upright accuracy	Simultaneous expression upright accuracy	0.31	0.147	0.456	< 0.001	0.0066	0.0001
Sorting expression upright accuracy	Simultaneous expression inverted accuracy	0.32	0.158	0.465	< 0.001	0.0066	0.0001
Sorting expression upright accuracy	Sorting identity upright accuracy	0.466	0.321	0.59	< 0.001	0.0066	0.0001
Sorting expression upright accuracy	Sorting identity inverted accuracy	0.355	0.197	0.495	< 0.001	0.0066	0.0001
Sorting expression upright accuracy	Sequential identity upright accuracy	0.383	0.228	0.52	< 0.001	0.0066	0.0001
Sorting expression upright accuracy	Sequential identity inverted accuracy	0.368	0.211	0.507	< 0.001	0.0066	0.0001
Sorting expression upright accuracy	Simultaneous identity upright accuracy	0.433	0.284	0.562	< 0.001	0.0066	0.0001
Sorting expression upright accuracy	Simultaneous identity inverted accuracy	0.383	0.228	0.519	< 0.001	0.0066	0.0001
Sorting expression inverted accuracy	Sequential expression upright accuracy	0.237	0.07	0.391	0.006	0.066	0.0069
Sorting expression inverted accuracy	Sequential expression inverted accuracy	0.315	0.153	0.461	< 0.001	0.0066	0.0001
Sorting expression inverted accuracy	Simultaneous expression upright accuracy	0.259	0.093	0.411	0.003	0.048	0.0037
Sorting expression inverted accuracy	Simultaneous expression inverted accuracy	0.365	0.208	0.504	< 0.001	0.0066	0.0001
Sorting expression inverted accuracy	Sorting identity upright accuracy	0.245	0.079	0.399	0.004	0.052	0.0049
Sorting expression inverted accuracy	Sorting identity inverted accuracy	0.345	0.186	0.487	< 0.001	0.0066	0.0001
Sorting expression inverted accuracy	Sequential identity upright accuracy	0.234	0.067	0.389	0.007	0.066	0.0077
Sorting expression inverted accuracy	Sequential identity inverted accuracy	0.298	0.135	0.446	< 0.001	0.0066	0.0001
Sorting expression inverted accuracy	Simultaneous identity upright accuracy	0.232	0.064	0.387	0.007	0.066	0.0077
Sorting expression inverted accuracy	Simultaneous identity inverted accuracy	0.281	0.117	0.431	0.001	0.019	0.0014
Sequential expression upright accuracy	Sequential expression inverted accuracy	0.713	0.618	0.788	< 0.001	0.0066	0.0001
Sequential expression upright accuracy	Simultaneous expression upright accuracy	0.42	0.269	0.551	< 0.001	0.0066	0.0001
Sequential expression upright accuracy	Simultaneous expression inverted accuracy	0.386	0.231	0.522	< 0.001	0.0066	0.0001
Sequential expression upright accuracy	Sorting identity upright accuracy	0.357	0.199	0.497	< 0.001	0.0066	0.0001
Sequential expression upright accuracy	Sorting identity inverted accuracy	0.24	0.072	0.394	0.005	0.06	0.006
Sequential expression upright accuracy	Sequential identity upright accuracy	0.484	0.342	0.605	< 0.001	0.0066	0.0001
Sequential expression upright accuracy	Sequential identity inverted accuracy	0.428	0.278	0.558	< 0.001	0.0066	0.0001
Sequential expression upright accuracy	Simultaneous identity upright accuracy	0.396	0.242	0.53	< 0.001	0.0066	0.0001
Sequential expression upright accuracy	Simultaneous identity inverted accuracy	0.297	0.134	0.445	< 0.001	0.0066	0.0001
Sequential expression inverted accuracy	Simultaneous expression upright accuracy	0.495	0.355	0.614	< 0.001	0.0066	0.0001
Sequential expression inverted accuracy	Simultaneous expression inverted accuracy	0.46	0.314	0.584	< 0.001	0.0066	0.0001
Sequential expression inverted accuracy	Sorting identity upright accuracy	0.288	0.124	0.437	< 0.001	0.0066	0.0001
Sequential expression inverted accuracy	Sorting identity inverted accuracy	0.304	0.141	0.45	< 0.001	0.0066	0.0001
Sequential expression inverted accuracy	Sequential identity upright accuracy	0.435	0.286	0.563	< 0.001	0.0066	0.0001
Sequential expression inverted accuracy	Sequential identity inverted accuracy	0.371	0.215	0.509	< 0.001	0.0066	0.0001
Sequential expression inverted accuracy	Simultaneous identity upright accuracy	0.487	0.346	0.607	< 0.001	0.0066	0.0001
Sequential expression inverted accuracy	Simultaneous identity inverted accuracy	0.402	0.249	0.535	< 0.001	0.0066	0.0001
Simultaneous expression upright accuracy	Simultaneous expression inverted accuracy	0.722	0.629	0.794	< 0.001	0.0066	0.0001
Simultaneous expression upright accuracy	Sorting identity upright accuracy	0.147	-0.024	0.309	0.092	0.184	0.0934
Simultaneous expression upright accuracy	Sorting identity inverted accuracy	0.265	0.099	0.416	0.002	0.036	0.0026
Simultaneous expression upright accuracy	Sequential identity upright accuracy	0.257	0.09	0.409	0.003	0.048	0.0037
Simultaneous expression upright accuracy	Sequential identity inverted accuracy	0.237	0.069	0.391	0.006	0.066	0.0069
Simultaneous expression upright accuracy	Simultaneous identity upright accuracy	0.498	0.358	0.616	< 0.001	0.0066	0.0001
Simultaneous expression upright accuracy	Simultaneous identity inverted accuracy	0.489	0.348	0.609	< 0.001	0.0066	0.0001
Simultaneous expression inverted accuracy	Sorting identity upright accuracy	0.111	-0.061	0.276	0.205	0.205	0.205
Simultaneous expression inverted accuracy	Sorting identity inverted accuracy	0.234	0.066	0.388	0.007	0.066	0.0077
Simultaneous expression inverted accuracy	Sequential identity upright accuracy	0.23	0.063	0.385	0.008	0.066	0.0085
Simultaneous expression inverted accuracy	Sequential identity inverted accuracy	0.187	0.017	0.346	0.031	0.124	0.0325
Simultaneous expression inverted accuracy	Simultaneous identity upright accuracy	0.395	0.241	0.529	< 0.001	0.0066	0.0001
Simultaneous expression inverted accuracy	Simultaneous identity inverted accuracy	0.378	0.222	0.515	< 0.001	0.0066	0.0001
Sorting identity upright accuracy	Sorting identity inverted accuracy	0.382	0.226	0.518	< 0.001	0.0066	0.0001
Sorting identity upright accuracy	Sequential identity upright accuracy	0.325	0.164	0.469	< 0.001	0.0066	0.0001
Sorting identity upright accuracy	Sequential identity inverted accuracy	0.286	0.121	0.435	< 0.001	0.0066	0.0001
Sorting identity upright accuracy	Simultaneous identity upright accuracy	0.302	0.139	0.449	< 0.001	0.0066	0.0001
Sorting identity upright accuracy	Simultaneous identity inverted accuracy	0.182	0.012	0.341	0.036	0.124	0.0371
Sorting identity inverted accuracy	Sequential identity upright accuracy	0.227	0.06	0.383	0.008	0.066	0.0085
Sorting identity inverted accuracy	Sequential identity inverted accuracy	0.303	0.14	0.45	< 0.001	0.0066	0.0001
Sorting identity inverted accuracy	Simultaneous identity upright accuracy	0.324	0.162	0.468	< 0.001	0.0066	0.0001
Sorting identity inverted accuracy	Simultaneous identity inverted accuracy	0.283	0.118	0.432	< 0.001	0.0066	0.0001
Sequential identity upright accuracy	Sequential identity inverted accuracy	0.628	0.512	0.721	< 0.001	0.0066	0.0001
Sequential identity upright accuracy	Simultaneous identity upright accuracy	0.394	0.24	0.529	< 0.001	0.0066	0.0001
Sequential identity upright accuracy	Simultaneous identity inverted accuracy	0.255	0.089	0.408	0.003	0.048	0.0037
Sequential identity inverted accuracy	Simultaneous identity upright accuracy	0.364	0.207	0.503	< 0.001	0.0066	0.0001
Sequential identity inverted accuracy	Simultaneous identity inverted accuracy	0.262	0.096	0.414	0.002	0.036	0.0026
Simultaneous identity upright accuracy	Simultaneous identity inverted accuracy	0.549	0.418	0.658	< 0.001	0.0066	0.0001

**Table 3:** Reaction time correlations between different conditions

Task 1	Task 2	Pearson’s r	Lower 95% CI	Upper 95% CI	p-value (uncorrected)	Holm p-value	Benjamini–Hochberg p-value
Sorting expression upright RT	Sorting expression inverted RT	0.926	0.898	0.947	< 0.001	< 0.001	< 0.001
Sorting expression upright RT	Sorting identity upright RT	0.841	0.783	0.885	< 0.001	< 0.001	< 0.001
Sorting expression upright RT	Sorting identity inverted RT	0.828	0.766	0.875	< 0.001	< 0.001	< 0.001
Sorting expression upright RT	Sequential expression upright RT	0.236	0.068	0.39	0.0063	0.0879	0.0078
Sorting expression upright RT	Sequential expression inverted RT	0.278	0.113	0.428	0.0012	0.0204	0.0016
Sorting expression upright RT	Sequential identity upright RT	0.198	0.028	0.356	0.0225	0.1714	0.0243
Sorting expression upright RT	Sequential identity inverted RT	0.212	0.043	0.369	0.0145	0.1449	0.0168
Sorting expression upright RT	Simultaneous expression upright RT	0.455	0.309	0.58	< 0.001	< 0.001	< 0.001
Sorting expression upright RT	Simultaneous expression inverted RT	0.465	0.32	0.588	< 0.001	< 0.001	< 0.001
Sorting expression upright RT	Simultaneous identity upright RT	0.378	0.222	0.515	< 0.001	0.0002	< 0.001
Sorting expression upright RT	Simultaneous identity inverted RT	0.483	0.34	0.603	< 0.001	< 0.001	< 0.001
Sorting expression inverted RT	Sorting identity upright RT	0.811	0.744	0.862	< 0.001	< 0.001	< 0.001
Sorting expression inverted RT	Sorting identity inverted RT	0.838	0.779	0.883	< 0.001	< 0.001	< 0.001
Sorting expression inverted RT	Sequential expression upright RT	0.175	0.005	0.335	0.044	0.1714	0.0454
Sorting expression inverted RT	Sequential expression inverted RT	0.226	0.058	0.382	0.0089	0.1153	0.0108
Sorting expression inverted RT	Sequential identity upright RT	0.151	-0.019	0.314	0.082	0.1714	0.082
Sorting expression inverted RT	Sequential identity inverted RT	0.162	-0.009	0.323	0.0628	0.1714	0.0638
Sorting expression inverted RT	Simultaneous expression upright RT	0.406	0.253	0.539	< 0.001	< 0.001	< 0.001
Sorting expression inverted RT	Simultaneous expression inverted RT	0.41	0.258	0.543	< 0.001	< 0.001	< 0.001
Sorting expression inverted RT	Simultaneous identity upright RT	0.339	0.179	0.482	< 0.001	0.0016	0.0001
Sorting expression inverted RT	Simultaneous identity inverted RT	0.441	0.292	0.568	< 0.001	< 0.001	< 0.001
Sorting identity upright RT	Sorting identity inverted RT	0.901	0.863	0.929	< 0.001	< 0.001	< 0.001
Sorting identity upright RT	Sequential expression upright RT	0.222	0.054	0.378	0.0101	0.1168	0.0119
Sorting identity upright RT	Sequential expression inverted RT	0.242	0.075	0.396	0.005	0.0756	0.0064
Sorting identity upright RT	Sequential identity upright RT	0.203	0.034	0.361	0.0193	0.1714	0.0215
Sorting identity upright RT	Sequential identity inverted RT	0.203	0.034	0.361	0.019	0.1714	0.0215
Sorting identity upright RT	Simultaneous expression upright RT	0.488	0.347	0.608	< 0.001	< 0.001	< 0.001
Sorting identity upright RT	Simultaneous expression inverted RT	0.457	0.311	0.582	< 0.001	< 0.001	< 0.001
Sorting identity upright RT	Simultaneous identity upright RT	0.42	0.269	0.551	< 0.001	< 0.001	< 0.001
Sorting identity upright RT	Simultaneous identity inverted RT	0.486	0.344	0.606	< 0.001	< 0.001	< 0.001
Sorting identity inverted RT	Sequential expression upright RT	0.188	0.018	0.347	0.0305	0.1714	0.0324
Sorting identity inverted RT	Sequential expression inverted RT	0.257	0.091	0.409	0.0028	0.0451	0.0037
Sorting identity inverted RT	Sequential identity upright RT	0.182	0.012	0.342	0.0359	0.1714	0.0376
Sorting identity inverted RT	Sequential identity inverted RT	0.199	0.03	0.357	0.0216	0.1714	0.0237
Sorting identity inverted RT	Simultaneous expression upright RT	0.437	0.288	0.565	< 0.001	< 0.001	< 0.001
Sorting identity inverted RT	Simultaneous expression inverted RT	0.437	0.288	0.565	< 0.001	< 0.001	< 0.001
Sorting identity inverted RT	Simultaneous identity upright RT	0.369	0.212	0.508	< 0.001	0.0003	< 0.001
Sorting identity inverted RT	Simultaneous identity inverted RT	0.461	0.315	0.585	< 0.001	< 0.001	< 0.001
Sequential expression upright RT	Sequential expression inverted RT	0.797	0.726	0.852	< 0.001	< 0.001	< 0.001
Sequential expression upright RT	Sequential identity upright RT	0.681	0.578	0.763	< 0.001	< 0.001	< 0.001
Sequential expression upright RT	Sequential identity inverted RT	0.727	0.636	0.799	< 0.001	< 0.001	< 0.001
Sequential expression upright RT	Simultaneous expression upright RT	0.415	0.264	0.547	< 0.001	< 0.001	< 0.001
Sequential expression upright RT	Simultaneous expression inverted RT	0.294	0.13	0.442	0.0006	0.0108	0.0008
Sequential expression upright RT	Simultaneous identity upright RT	0.328	0.167	0.472	0.0001	0.0026	0.0002
Sequential expression upright RT	Simultaneous identity inverted RT	0.223	0.055	0.379	0.0097	0.1168	0.0117
Sequential expression inverted RT	Sequential identity upright RT	0.705	0.608	0.782	< 0.001	< 0.001	< 0.001
Sequential expression inverted RT	Sequential identity inverted RT	0.764	0.683	0.827	< 0.001	< 0.001	< 0.001
Sequential expression inverted RT	Simultaneous expression upright RT	0.466	0.321	0.59	< 0.001	< 0.001	< 0.001
Sequential expression inverted RT	Simultaneous expression inverted RT	0.352	0.193	0.492	< 0.001	0.0009	0.0001
Sequential expression inverted RT	Simultaneous identity upright RT	0.347	0.188	0.489	< 0.001	0.0011	0.0001
Sequential expression inverted RT	Simultaneous identity inverted RT	0.329	0.169	0.473	0.0001	0.0025	0.0002
Sequential identity upright RT	Sequential identity inverted RT	0.805	0.736	0.858	< 0.001	< 0.001	< 0.001
Sequential identity upright RT	Simultaneous expression upright RT	0.392	0.238	0.527	< 0.001	0.0001	< 0.001
Sequential identity upright RT	Simultaneous expression inverted RT	0.312	0.15	0.458	0.0003	0.0051	0.0004
Sequential identity upright RT	Simultaneous identity upright RT	0.395	0.24	0.529	< 0.001	0.0001	< 0.001
Sequential identity upright RT	Simultaneous identity inverted RT	0.323	0.162	0.468	0.0001	0.0031	0.0002
Sequential identity inverted RT	Simultaneous expression upright RT	0.425	0.274	0.555	< 0.001	< 0.001	< 0.001
Sequential identity inverted RT	Simultaneous expression inverted RT	0.299	0.136	0.446	0.0005	0.009	0.0007
Sequential identity inverted RT	Simultaneous identity upright RT	0.449	0.301	0.575	< 0.001	< 0.001	< 0.001
Sequential identity inverted RT	Simultaneous identity inverted RT	0.339	0.179	0.481	0.0001	0.0016	0.0001
Simultaneous expression upright RT	Simultaneous expression inverted RT	0.812	0.745	0.863	< 0.001	< 0.001	< 0.001
Simultaneous expression upright RT	Simultaneous identity upright RT	0.625	0.509	0.719	< 0.001	< 0.001	< 0.001
Simultaneous expression upright RT	Simultaneous identity inverted RT	0.665	0.557	0.75	< 0.001	< 0.001	< 0.001
Simultaneous expression inverted RT	Simultaneous identity upright RT	0.565	0.437	0.671	< 0.001	< 0.001	< 0.001
Simultaneous expression inverted RT	Simultaneous identity inverted RT	0.621	0.504	0.716	< 0.001	< 0.001	< 0.001
Simultaneous identity upright RT	Simultaneous identity inverted RT	0.722	0.629	0.795	< 0.001	< 0.001	< 0.001

## Footnotes

1 The authors used three kinds of tasks: simultaneous matching, sequential matching, and sorting. Each task had two versions – identity (focusing on recognizing individuals) and expression (focusing on recognizing emotions) – and was performed in both upright and inverted orientations. In Tasks 1 and 2 participants were instructed to respond at their own pace.

- Simultaneous Matching Task: Participants were shown three faces at the same time and had to identify the odd one out based on either identity or expression. In the identity task, two faces belonged to the same person, while the third was a different individual. In the expression task, two faces displayed the same emotion, while the third showed a different one.
- Sequential Matching Task: Participants viewed two faces in succession and judged whether they showed the same or different identity/expression. The first face appeared briefly, followed by a short interval, and then the second face was presented. In the identity task, expressions varied, requiring participants to focus on identity. In the expression task, identities varied, requiring participants to focus on emotion.
- Sorting Task: Participants arranged six faces in order of similarity to a target face, based on either identity or expression. In the identity task, they sorted morphs of a person’s face. In the expression task, they arranged morphs that varied in intensity of a given emotion.

For the simultaneous and sequential matching tasks, upright and inverted trials were presented in separate blocks, whereas in the sorting task, orientations were intermixed. All tasks were administered online using Testable and raw data are available on the Open Science Framework (OSF) (https://osf.io/uwk9y/).

## Code availability

All analysis coding is available on the OSF.
